# Mutant TDP-43 drives impairments in axonal transport and glycolysis in a mouse stem-cell-derived motor neuron model of amyotrophic lateral sclerosis (ALS)

**DOI:** 10.1038/s41419-026-08437-2

**Published:** 2026-01-31

**Authors:** Emily Carroll, Jakub Scaber, Iris-Stefania Pasniceanu, Ruxandra Dafinca, David Gordon, Ana Candalija, Kevin Talbot

**Affiliations:** 1 Nuffield Department of Clinical Neurosciences, University of Oxford, Oxford, UK; 2 Kavli Institute for Nanoscience Discovery, Dorothy Crowfoot Hodgkin Building, Oxford, UK

**Keywords:** Neuroscience, Diseases of the nervous system, Amyotrophic lateral sclerosis

## Abstract

TDP-43 dysfunction is thought to be central to ALS pathogenesis. Studying mutations in the gene which encodes TDP-43, *TARDBP*, provides a valuable opportunity to gain insight into how TDP-43 dysfunction alters cellular homoeostasis. Our group has previously developed a TDP-43^M337V^ mouse embryonic stem cell-derived motor neuron (mESC-MN) model, which expresses a single copy of the human *TARDBP* gene expressing the pathogenic M337V mutation at low levels. Here, we perform extensive phenotypic characterisation of this model, and show that TDP-43^M337V^ leads to reduced MN viability, impaired axonal transport and reduced basal glycolysis compared to TDP-43^WT^ controls. Altered neuronal viability and function occurs in the absence of TDP-43 mislocalisation or aggregation, suggesting ‘proteinopathy’ is downstream of these ALS-relevant phenotypes. These findings provide further support for a link between TDP-43 dyshomeostasis, cellular bioenergetics and axonal transport and suggest these pathways warrant further investigation as targets for therapeutic intervention.

## Introduction

Amyotrophic lateral sclerosis (ALS) is the most common form of motor neuron disease (MND), resulting from the degeneration of motor neurons in the motor cortex, brainstem and spinal cord, leading to progressive paralysis [1]. Disturbances in a range of cellular and molecular processes have been consistently observed in autopsy specimens, and in cellular and animal models, and these are reviewed in detail elsewhere [2]. However, the relative contribution of each of these mechanisms and the temporal sequence of events culminating in motor neuron loss in ALS remains to be elucidated.

Amid this complexity, TDP-43 dysfunction is a unifying feature observed in the vast majority of ALS cases and is thought to be central to disease pathogenesis. TDP-43 is an RNA-binding protein with important roles in RNA metabolism and in regulating gene transcription [3]. TDP-43 also binds to other proteins and, in doing so, regulates many important cellular functions crucial for overall cell health [4–7]. In CNS tissue from patients who have died of ALS or frontotemporal dementia (FTD), TDP-43 is found to mislocalise to the cytoplasm from its normally nuclear location, leading to loss of nuclear function and the formation of potentially toxic cytoplasmic aggregates [8, 9]. Mutations in the gene which encode TDP-43, *TARDBP*, have been observed in a minority of ALS cases, providing evidence of a direct mechanistic link between TDP-43 dysfunction and disease [10]. Mutations in TDP-43 are preferentially localised to the C-terminal glycine-rich domain (GRD) [11]. The GRD is the largest and most crucial domain underlying the ability of TDP-43 to bind to other proteins, suggesting loss of this capability may be an important aspect of TDP-43 function underlying ALS pathogenesis [12].

We previously developed in vivo and in vitro mouse models of the TDP-43^M337V^ pathogenic mutation [13, 14]. These models contain a stably integrated single copy of a bacterial artificial chromosome (BAC) construct which carries the entire genomic locus of the wild-type or M337V mutant human *TARDBP* gene. Expression of this construct leads to the production of human TDP-43^WT^ or TDP-43^M337V^ protein at low levels on a background of endogenous mouse Tdp-43 expression. A Ypet tag allows identification of the human exogenous TDP-43 protein product. Expression of TDP-43^M337V^ in BAC transgenic mice leads to progressive loss of motor function, loss of neuromuscular junction integrity, impaired retrograde axonal transport of signalling endosomes and reduced survival [14, 15]. In primary motor neurons, extracted from the spinal cords of embryonic mice, TDP-43^M337V^ expression leads to cytoplasmic mislocalisation of TDP-43 and impaired stress granule formation [14]. Similarly, TDP-43^M337V^ mouse embryonic stem cell-derived motor neurons (mESC-MNs) show impaired responses to oxidative stress, including reduced viability and impaired stress granule formation [14].

Here, we characterise the effects of the TDP-43^M337V^ pathogenic mutation in unstressed conditions across an extended range of cellular phenotypes in our mESC-MNs model, with the aim of gaining greater insight into the early drivers of cellular dysfunction. We identify several phenotypes in TDP-43^M337V^ mESC-MNs, including reduced viability, reduced speed of axonal transport and impaired glycolysis.

## Methods

### Mouse embryonic stem cell-derived motor neuron (mESC-MN) culture

**Fig. 1:**
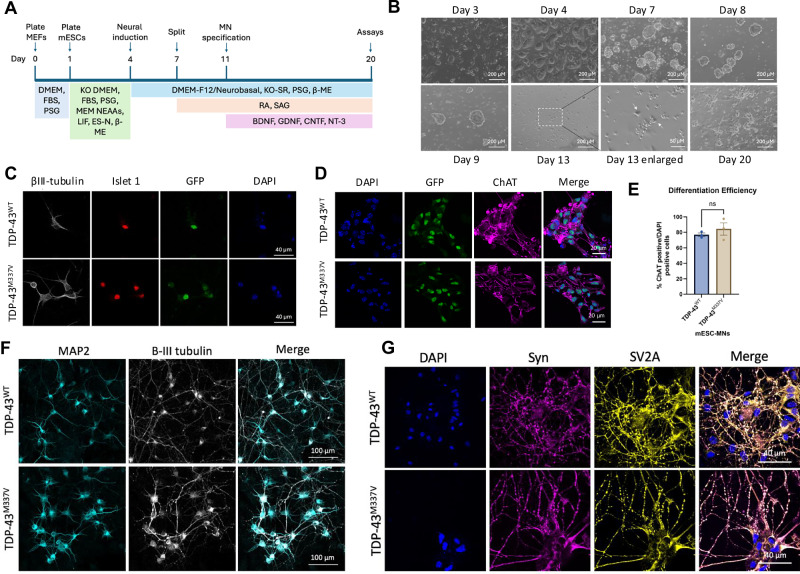
TDP-43^M337V^ mESCs can be differentiated into motor neurons in vitro. **A** Protocol for the differentiation of mESCs to motor neurons. mESCs are plated onto a bed of mouse embryonic fibroblasts (MEFs) in Knockout DMEM (KO DMEM) supplemented with foetal bovine serum (FBS) and penicillin/streptomycin/glutamine (PSG), MEM non-essential amino acids (MEM NEAAs), leukaemia inhibitory factor (LIF), EmbryoMax embryonic stem cell qualified nucleotides (ES-N) and beta mercaptoethanol (β-ME). Neural induction is started on day 4, with DMEM-F12, Neurobasal, knock-out serum replacement (KO SR), PSG and β-ME. Embryoid bodies were split on day 7 and retinoic acid (RA) and smoothened agonist (SAG) added to the media. Motor neuron specification was started on day 11 with the addition of growth factors including BDNF, GDNF, CNTF and NT-3. **B** TDP-43^WT^ mESCs at different stages of the differentiation protocol. The arrows in ‘day 13 enlarged’ indicate neuronal cell bodies that are beginning to extend processes. **C** mESC-MNs express neuronal markers, including islet 1 and β-III tubulin and GFP detecting human exogenous TDP-43 carried by the BAC construct by day 14. **D** mESC express the motor neuron marker choline acetyltransferase (ChAT) by day 14. **E** Differentiation efficiency is approximately 80% for both TDP-43^WT^ and TDP-43^M337V^ mESC-MNs at day 14. *N* = 3 differentiations. Error bars = SEM. **F** TDP-43^WT^ and TDP-43^M337V^ express MAP2 and β-III tubulin at day 20. **G** TDP-43^WT^ and TDP-43^M337V^ express the synaptic markers synaptophysin (Syn) and SV2A at Day 20.

mESCs were generated by the Wellcome Trust Centre for Human Genetics, University of Oxford [14]. mESCs were differentiated to motor neurons as previously described [14, 16] and detailed in Fig. 1. In brief, mESCs were plated on primary mouse embryonic fibroblasts, then lifted and expanded as embryoid bodies (EBs), in ADFNK media supplemented with retinoic acid (RA) and smoothened agonist (SAG). EBs were then dissociated and seeded into poly-l-ornithine (Sigma, 1:10 in sterile water) and mouse laminin (2.5 μg/ml in HBSS) coated plates as a single cell suspension at the required density. ADFNK media was supplemented with growth factors (10 ng/ml glia-derived neurotrophic factor (GDNF), 10 ng/ml BDNF, 25 ng/ml CNTF and 10 ng/ml NT-3; all from PeproTech) to induce motor neuron specification. mESCs were tested for mycoplasma using the MycoAlert® Mycoplasma Detection Kit (Lonza; LT07-318).

### Viability analysis

Cellular viability at day 20 was analysed using the CellTiter 96^®^ AQ_ueous_ One Solution Cell Proliferation Assay (Promega). Cells were incubated in 100 μL of normal culture medium per well together with 20 μL of CellTiter 96^®^ AQ_ueous_ One Solution Reagent at 37 °C, 5% CO_2_ for 3 h. Absorbance was read at 490 nm using a plate reader.

### Protein extraction and Western blotting

Cells were lysed in RIPA buffer and mixed with NuPAGE LDS sample buffer and NuPAGE sample reducing agent and heated at 95 °C for 5 min. Resolved protein lysates were separated by SDS-PAGE using 4–12% Bis-Tris pre-cast gels (Life Technologies) (10 μg of protein loaded per well), then transferred to a polyvinylidene difluoride membrane using the iBlot 2 Gel Transfer Device (ThermoFisher) for blocking in 1X Tris-buffered saline (TBS) containing 0.1% Tween-20 and 5% skimmed milk. Following blocking, primary antibodies were incubated in 1X TBS with 0.1% Tween-20 and 1% skimmed milk. Primary antibodies used were *pan*TDP-43 (Proteintech; 10782-2-AP; 1:1000), dynactin 1 (Invitrogen; PA5-21289; 1:1000), dynein (Abcam; ab23905; 1:1000), KIF5A (Abcam; ab5628; 1:1000), KIF5B (Abcam; ab25715; 1:1000), KIF5ABC (Abcam; ab271049; 1:1000), TOMM20 (Abcam; ab186735; 1:1000), COX IV (Abcam; ab14744; 1:1000), OPA1 (BD Biosciences; 612606; 1:500) and β-actin (Abcam; ab227387; 1:1000) as a loading control. Blots were washed three times in PBS and incubated with horseradish peroxidase (HRP)-conjugated secondary antibodies (BioRad;1:5000) in 1X TBS with 0.1% Tween-20 and 1% skimmed milk for 1 h at room temperature. Blots were washed and imaged using the Bio-Rad ChemDoc. Integrated optical density of the protein bands was analysed in Fiji/ImageJ and normalised to β-actin from the same blot. Full and uncropped Western blots can be found in the Supplementary Material.

### Immunocytochemistry

Motor neurons were fixed in 4% paraformaldehyde (PFA)-PBS for 20 min. Samples were blocked and permeabilised for 1 h at room temperature in blocking buffer (0.1% Triton X-100, 1X PBS) supplemented with 5% normal donkey or goat serum. Samples were incubated overnight at 4°C with primary antibodies diluted in blocking buffer supplemented with 1% donkey or goat serum. Coverslips were incubated with fluorescently labelled secondary antibodies raised in donkey or goat; AlexaFluor®488; AlexaFluor®568, AlexaFluor®647 (Life Technologies; 1:500). Secondary antibodies were added for 1 h at room temperature. DAPI was included during washing in PBS (1:10,000). Mounting media was not required for microscopy of microfluidic chambers, which were stored in PBS following immunocytochemistry until visualisation. Coverslips were mounted in ProLong^TM^ Glass Antifade Mountant (Thermo Fisher Scientific) for visualisation. To stain mitochondria in living cells, MitoTracker^TM^ Red CMXRos (Thermo Fisher Scientific; M7512) was prepared at 100 nM in growth medium and incubated for 45 min at 37 °C prior to fixation. Antibodies used included: Islet 1 (DSHB; 40.2D6-A; 1:100), ChAT (Millipore; ab144P; 1:100), *pan*TDP-43 (Proteintech; 10782-2-AP; 1:1000), GFP (Invitrogen; 10474172; 1:1000), SV2A (Synaptic Systems; 119002; 1:200), synaptophysin (abcam; ab52636; 1:1000), β-III tubulin (Novus Biologicals;NB100-1612; 1:100), TAU (Millipore; MAB3420; 1:500), MAP2 (Millipore; ab5622; 1:1000) and TOMM20 (Abcam; ab186735; 1:1000).

### Confocal microscopy and image analysis

Samples were imaged using a Zeiss Airyscan confocal microscope. Images were acquired with a 63X objective and 10.24 resolution. Samples were blinded prior to imaging and analysis. Confocal images were analysed using Fiji/ImageJ software. A minimum of 5 images was analysed per genotype and per differentiation, for at least three differentiations. The integrated density was calculated to quantify the fluorescence intensity of TDP-43. Nuclear and cytoplasmic regions were identified using DAPI and ChAT staining, respectively. The percentage of cytoplasmic TDP-43 was calculated as the integrated density for the cytoplasmic region divided by the integrated density for nuclear and cytoplasmic regions. The neurite area was quantified as the area of β-III tubulin per field of view. MitoTracker intensity was calculated as the integrated density of MitoTracker divided by the integrated density of TOMM20. To calculate mitochondria circularity and aspect ratio, images were analysed in Fiji/ImageJ using the tubeness plugin followed by thresholding. The resultant image was the analysed to calculate mitochondrial descriptors, including circularity, major axis and minor axis. The mitochondria aspect ratio was calculated as the major axis divided by the minor axis.

### Axonal transport analysis

Microfluidic chambers (Xona, RD450) were plasma-bonded to glass-bottomed dishes (Wilco) by a 2-min treatment at 300–500 millitorr. Devices were coated in poly-l-ornathine (Sigma, 1:10 in sterile water) overnight at 4 °C, then washed in sterile water and coated in laminin (2.5 μg/mL in HBSS) overnight at 4 °C. On day 11, mESC-MN precursors were plated into the top left-hand side of the microfluidic chambers at a density of approximately 100,000 cells per chamber in 5 μL growth media. Media on the right-hand side of the chamber was supplemented with growth factors (BDNF, GDNF, CNTF, and NT-3) at 10× higher concentration (100 ng/mL), to encourage axons to extend through the channels. Chambers were incubated at 37 °C and 5% CO_2_ for a further 9 days prior to analysis. Mitochondrial transport was assessed using MitoTracker^TM^ Red CMXRos (M7512) and the retrograde tracer cholera toxin subunit-B (CTB) AlexaFluor^®^ 488 conjugate (Invitrogen, C34775) was used to investigate transport of CTB positive signalling endosomes. Cells were treated with MitoTracker^TM^ Red CMXRos at 20 nM in normal growth media, which was added to both sides of the chamber for 20 min at 37 °C. Cells were treated with CTB AlexaFluor^®^ 488 at 100 ng/mL in normal growth media, which was added to the axonal side of the microfluidic chamber for 10 min at 37 °C. Live imaging was performed within the microgrooves of the microfluidic chamber using the Olympus SoRa spinning-disk confocal microscope at 63× magnification in the confocal setting. All live imaging was performed at 37 °C and 5% CO_2_. Analysis was performed using the difference tracker Fiji/Image J plugin developed by Andrews and colleagues [17] and subsequently in R using a script developed by Dr Jakub Scaber, which is available at http://github.com/jscaber/sensorimotor/tree/main/live [18].

### Seahorse assays

The Seahorse Bioscience Extracellular Flux Analyzer (XF96, Seahorse Bioscience Inc., North Billerica, MA, USA) was used to investigate cellular bioenergetics in mESC-MNs. For both the Seahorse XF Cell Mito Stress Test (Agilent) and Glycolytic Rate Assay Seahorse XF Glycolytic Rate Assay (Agilent), cells were plated in a 96-well Seahorse microplate (Agilent) at a density of 100,000 cells/well in Seahorse XF DMEM, supplemented with 1 mM pyruvate, 2 mM glutamine and 10 mM glucose. Motor neurons were then incubated in 180 μL Seahorse XF DMEM at 37 °C, no CO_2_ for 45 min. For the Mito Stress Test, compounds were used at a final concentration of 1.5 μM of oligomycin, 1 μM of FCCP and 0.5 μM of Rotenone/Antimycin A. For the Glycolytic Rate Assay, Rotenone/Antimycin A and 2-deoxy-D-glucose (2-DG) were used at a final concentration of 0.5 μM and 50 μM, respectively. Oxygen consumption rates (OCR) and proton efflux rates (PER) were normalised by cell number per well using the CyQuant Cell Proliferation Assay (Thermo Fisher Scientific). Microplates were frozen at −80 °C, then thawed at room temperature and cell count was determined using the CyQUANT^®^ Cell Proliferation Assay (Thermo Fisher Scientific). The cell-lysis buffer stock solution (20×) was diluted in distilled water and the CyQUANT^®^ GR stock solution was diluted 400-fold into the 1X cell-lysis buffer. 200 μL of the CyQUANT^®^ GR dye/cell-lysis buffer was added to each sample well and incubated at room temperature for 10 min protected from light. Sample fluorescence was measured using a fluorescence microplate reader with filters appropriate for 480 nm excitation and 520 nm emission.

### Statistical analysis

Data are represented as mean ± SEM. *p* values were calculated using two-tailed unpaired *t*-tests (or non-parametric Mann-Whitney *U* test). Statistical significance was considered *p* < 0.05. For all experiments involving mESC-MNs, a minimum of 3*n* biological replicates were included unless otherwise stated. Centre values in graphs indicate the mean. Error bars represent the standard error of the mean (SEM). For all ICC experiments, samples were blinded prior to analysis. It was not possible to blind results for the Seahorse or axonal transport analysis; however, automated, unbiased analysis pipelines were used for quantification.

## Results

### mESCs express human TDP-43^WT^ or TDP-43^M337V^ at low levels and differentiate to form motor neurons

mESCs were differentiated to motor neurons based on previously published protocols [14, 19, 20], and directed towards a motor neuron cell fate through the addition of retinoic acid (RA), smoothened agonist (SAG) and a cocktail of neurotrophic factors (Fig. 1A). mESCs were differentiated as 3D embryoid bodies, which were dissociated on day 11 and seeded as a single cell suspension. mESC-MNs begin to develop processes one day after seeding and form networks of neurons following a further 2 days in vitro (Fig. 1B). mESC-MNs were maintained up to 20 days in vitro prior to performing phenotypic analysis.

mESC-MNs express the neuronal marker β-III tubulin and the motor neuron markers islet 1 and choline acetyltransferase (ChAT) (Fig. 1C, D). mESC-MNs also express the Ypet tag (detected as GFP) carried by the BAC construct (Fig. 1C, D). Differentiation efficiency, determined by the number of ChAT-positive cells divided by the number of DAPI-positive nuclei at day 14, is approximately 80% and does not significantly vary between TDP-43^WT^ and TDP-43^M337V^ mESC-MNs (*p* = 0.4140) (Fig. 1E). At day 20, mESC-MNs express mature neuronal markers, including MAP2 and the synaptic markers SV2A and synaptophysin (Fig. 1F, G). DNA extracted from mESC-MNs was amplified with PCR and examined by gel electrophoresis to confirm expression of the exogenous human TDP-43 and the Ypet tag carried by the BAC construct (Supplementary Fig. 1A). DNA sequencing confirmed expression of the M337V substitution mutation in TDP-43^M337V^ mESC-MNs; a single base pair change from adenine (A) to guanine (G) within exon 6 of the *TARDBP* gene (Supplementary Fig. 1B). Western blotting confirmed the expression of the human TDP-43 protein in TDP-43^WT^ and TDP-43^M337V^ mESC-MNs compared to non-transgenic (NTg) control mESC-MNs (Supplementary Fig. 1C). Expression of the exogenous human TDP-43 protein occurred at low levels (40–60%) relative to the mouse endogenous TDP-43 protein. The expression ratio between human TDP-43 and mouse TDP-43 was not significantly different between TDP-43^WT^ and TDP-43^M337V^ mESC-MNs (Supplementary Fig. 1D).

### TDP-43^M337V^ mESC-MNs show reduced viability, without evidence of TDP-43 mislocalisation or aggregation

TDP-43 mislocalisation, from its normally nuclear location to the cytoplasm, is the pathological hallmark of ALS. Previous work has demonstrated that overexpression of TDP-43^M337V^ from this BAC construct in HEK cells leads to significantly increased cytoplasmic localisation of GFP-TDP-43 and reduced cell viability, compared to TDP-43^WT^ and non-transgenic controls [13].

**Fig. 2:**
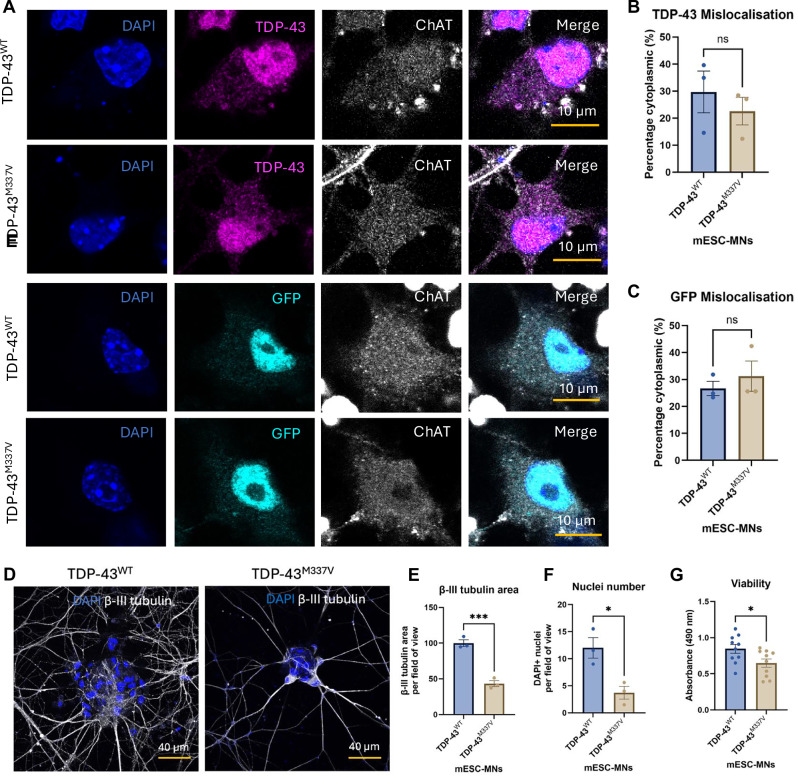
TDP-43^M337V^ mESC-MNs show reduced viability without evidence of TDP-43 mislocalisation or aggregation. **A** Immunocytochemistry for total cellular TDP-43 and human exogenous TDP-43, the latter detected by GFP to label the Ypet tag carried by the BAC construct. **B** Quantification of the percentage of total cellular cytoplasmic TDP-43 in TDP-43^WT^ and TDP-43^M337V^ mESC-MNs. *N* = 3 differentiations. **C** Quantification of the percentage of cytoplasmic GFP in TDP-43^WT^ and TDP-43^M337V^ mESC-MNs. *N* = 3 differentiations. **D** β-III tubulin in TDP-43^WT^ and TDP-43^M337V^ mESC-MNs at day 20. **E** Neurite area per field of view in TDP-43^WT^ and TDP-43^M337V^ mESC-MNs quantified as β-III tubulin area *N* = 3. **F** Nuclei number in TDP-43^WT^ and TDP-43^M337V^ mESC-MNs quantified using DAPI. *N* = 3 differentiations. **G** TDP-43^M337V^ mESC-MNs show reduced viability compared to TDP-43^WT^ controls. *N* = 10 differentiations. *p = <0.05. Error bars = SEM.

To investigate whether expression of the TDP-43^M337V^ mutation leads to increased cytoplasmic localisation of TDP-43 in mESC-MNs, immunocytochemistry was performed (Fig. 2A). Antibodies for GFP and TDP-43 were used to investigate the cellular localisation of human TDP-43 and total cellular TDP-43, respectively. No significant differences in TDP-43 or GFP mislocalisation were identified between TDP-43^WT^ and TDP-43^M337V^ mESC-MNs (TDP-43: *p* = 0.4851; GFP: *p* = 0.5049), with cytoplasmic TDP-43 and GFP accounting for ~20–30% of total cellular TDP-43 and GFP (Fig. 2B, C).

To investigate whether expression of TDP-43^M337V^ alters cellular viability in the mESC-MN model, we examined neurite area and cellular viability in mESC-MNs at day 20. Analysis of β-III tubulin revealed significantly reduced neurite area in TDP-43^M337V^ mESC-MNs compared to TDP-43^WT^ controls (*p* = 0.0009), accompanied by a significant reduction in nuclei number (*p* = 0.0279) (Fig. 2D-F). In support of this, and in line with observations from TDP-43^M337V^ HEK cells, TDP-43^M337V^ mESC-MNs showed significantly reduced viability measured using the MTS assay compared to TDP-43^WT^ controls (*p* = 0.0313) (Fig. 2G).

### TDP-43^M337V^ mESC-MNs show impaired axonal transport of CTB-positive signalling endosomes and mitochondria

**Fig. 3:**
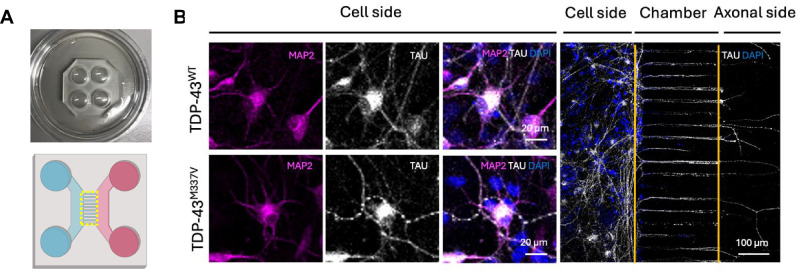
Analysis of axonal transport in mESC-MNs using microfluidic chambers. **A** Microfluidic chambers containing microgrooves were used for the assessment of axonal transport. The yellow dotted line indicates the microgrooves within the chamber where live imaging was performed. **B** Immunocytochemistry for the dendritic marker MAP2, and the axonal marker TAU to confirm axonal identity of processes extending through the microgrooves.

Impaired axonal transport represents a shared pathophysiological feature observed across genetically distinct models of the disease, including models of *SOD1*, *C9ORF72*, *FUS*, and *TARDBP* mutations [21–26]. In previous work, our TDP-43^M337V^ BAC transgenic mice showed significantly impaired retrograde transport of neurotrophin-containing signalling endosomes in vivo, [27] however, axonal transport in our mESC-MN model has not previously been investigated. To investigate axonal transport in mESC-MNs, silicone microfluidic chambers were used (Fig. 3A). Staining for the axonal marker TAU and the dendritic marker MAP2 confirmed the axonal identity of processes extending across the microgrooves (Fig. 3B). To investigate the axonal transport of CTB positive signalling endosomes, chambers were incubated with the retrograde tracer, cholera toxin B (CTB) [28]. To investigate the axonal transport of mitochondria, chambers were incubated with MitoTracker. Live imaging was performed to investigate the retrograde transport of CTB and the bidirectional transport of mitochondria.

**Fig. 4:**
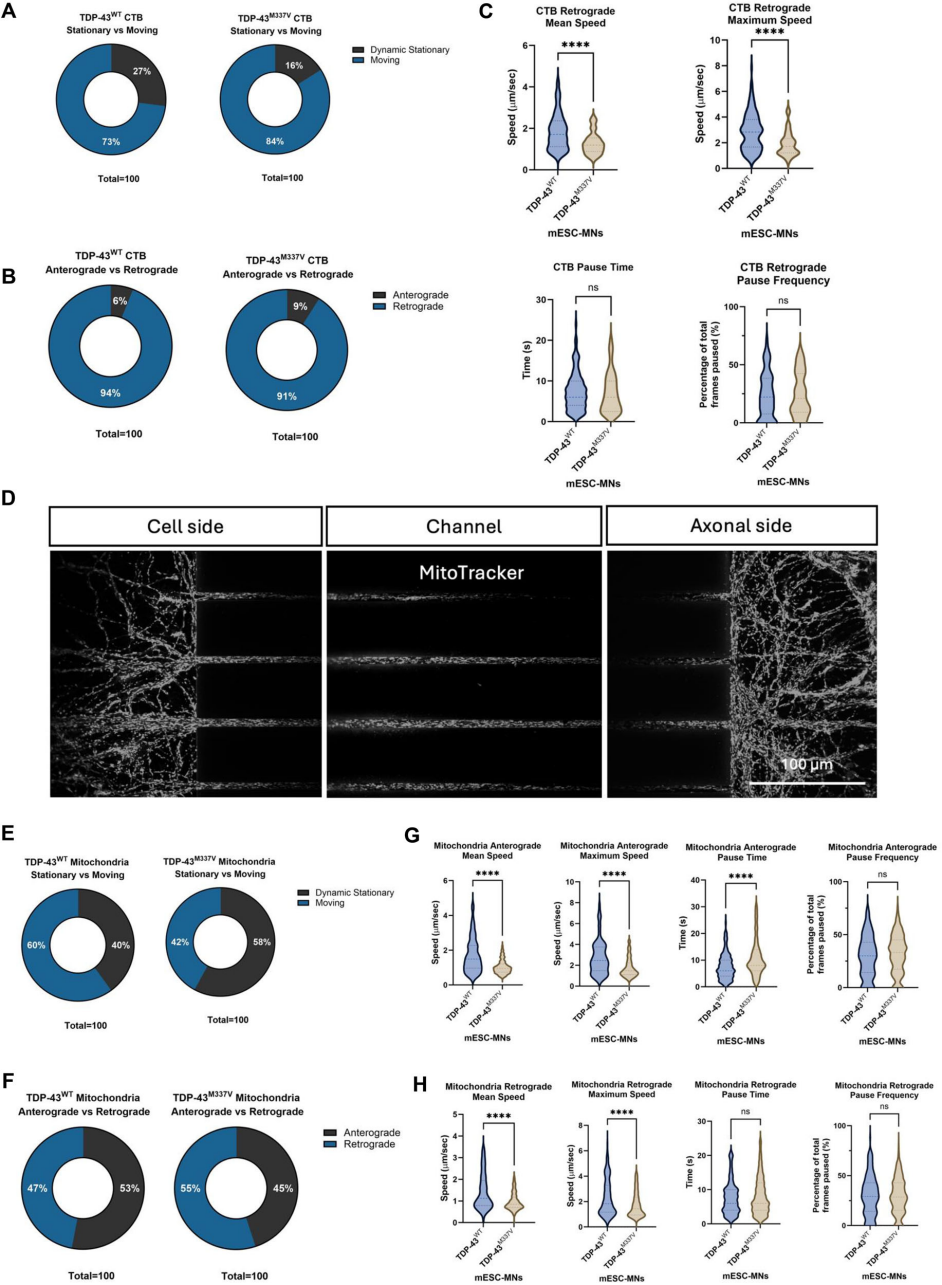
TDP-43^M337V^ mESC-MNs show impaired axonal transport of CTB-positive signalling endosomes and mitochondria. **A** The percentage of signalling endosomes that were moving or dynamically stationary in TDP-43^WT^ and TDP-43^M337V^ mESC-MNs. **B** The percentage of signalling endosomes moving in the anterograde versus retrograde direction in TDP-43^WT^ and TDP-43^M337V^ mESC-MNs. **C** Quantification of the mean speed, maximum speed, pause time and pause frequency of signalling endosomes in TDP-43^WT^ and TDP-43^M337V^ mESC-MNs. **D** MitoTracker staining to identify mitochondria in microfluidic chambers. **E** The percentage of mitochondria that were moving or dynamic stationary in TDP-43^WT^ and TDP-43^M337V^ mESC-MNs. **F** The percentage of mitochondria moving in the anterograde versus retrograde direction in TDP-43^WT^ and TDP-43^M337V^ mESC-MNs. **G** Quantification of the mean speed, maximum speed, pause time and pause frequency of mitochondria moving in the anterograde direction in TDP-43^WT^ and TDP-43^M337V^ mESC-MNs. **H** Quantification of the mean speed, maximum speed, pause time and pause frequency of mitochondria moving in the retrograde direction in TDP-43^WT^ and TDP-43^M337V^ mESC-MNs. For CTB experiments: TDP-43^WT^ *N* = 5 differentiations, TDP-43^M337V^ *N* = 3 differentiations. For MitoTracker experiments: *N* = 6 differentiations for TDP-43^WT^ and *N* = 7 differentiations for TDP-43^M337V^. **p = <0.01, ****p = <0.0001. Error bars = SEM.

Analysis of CTB axonal transport revealed that 73% and 84% of the CTB positive signalling endosomes detected were moving in TDP-43^WT^ and TDP-43^M337V^ mESC-MNs respectively (Fig. 4A). The majority of CTB positive signalling endosomes (94% for TDP-43^WT^ and 91% for TDP-43^M337V^ mESC-MNs) were transported in the retrograde direction as expected (Fig. 4B). Analysis of CTB positive signalling endosomes moving in the retrograde direction revealed significantly reduced mean speed (*p* ≤0.0001) and maximum speed (*p* ≤ 0.0001) of movement in TDP-43^M337V^ mESC-MNs compared to TDP-43^WT^ controls. No significant differences were observed in the pause time (*p* = 0.4703) or pause frequency (*p* = 0.7134) of CTB positive signalling endosomes between TDP-43^WT^ and TDP-43^M337V^ mESC-MNs (Fig. 4C).

Analysis of mitochondrial transport identified bi-directional movement of mitochondria in TDP-43^WT^ and TDP-43^M337V^ mESC-MNs (Fig. 4D). The percentage of moving mitochondria was 60% in TDP-43^WT^ mESC-MNs compared with 42% for TDP-43^M337V^ mESC-MNs, with 40% and 58% of mitochondria classed as dynamic stationary in TDP-43^WT^ and TDP-43^M337V^ mESC-MNs respectively (Fig. 4E). In TDP-43^WT^ mESC-MNs, 53% of mitochondria were moving in the anterograde direction with 47% of mitochondria moving in the retrograde direction. In TDP-43^M337V^ mESC-MNs, 45% of mitochondria were moving in the anterograde direction with 55% of mitochondria moving in the retrograde direction (Fig. 4F). The speed of mitochondrial transport was significantly reduced in TDP-43^M337V^ mESC-MNs compared to TDP-43^WT^ controls in both the anterograde (mean speed: *p* = <0.0001, max speed: *p* = <0.0001) and retrograde direction (mean speed: *p* = <0.0001, max speed: *p* = <0.0001) (Fig. 4G, H). For mitochondria moving in the anterograde direction, the time spent in pause was significantly increased in TDP-43^M337V^ mESC-MNs compared to TDP-43^WT^ controls (*p* = 0.0054), although there were no differences in pause frequency (*p* = 0.1615) (Fig. 4G). No significant differences in pause time (*p* = 0.2586) or pause frequency (*p* = 0.9418) were identified between TDP-43^WT^ and TDP-43^M337V^ mESC-MNs for mitochondria moving retrogradely (Fig. 4H).

### The neuronal cytoskeleton and axonal transport machinery are not significantly altered in TDP-43^M337V^ mESC-MNs

The neuronal cytoskeleton is essential for maintaining proper cellular function through segregating the somatic, axonal and synaptic compartments. The cytoskeleton is comprised of actin filaments, neurofilaments and microtubules. Reduced axon formation and axon length have been observed in cellular and animal models of ALS expressing mutant forms of TDP-43, suggesting that TDP-43 dysfunction may play an important in the maintenance of the axonal cytoskeleton [16, 29–31]. In Fig. 2, we observed a significant reduction in area of the microtubule marker β-III tubulin in TDP-43^M337V^ mESC-MNs compared to TDP-43^WT^ controls (Fig. 2E). To investigate whether the expression of molecular motor proteins is altered in TDP-43^M337V^ mESC-MNs, the expression of the anterograde motor proteins, kinesins, and retrograde motor proteins, dynein and dynactin, were assessed in protein samples collected from mass culture. No significant differences in the expression of molecular motor proteins were identified between TDP-43^WT^ and TDP-43^M337V^ mESC-MNs (*p* > 0.05) (Supplementary Fig. 2).

### Impaired glycolysis, but not mitochondrial respiration, is observed in TDP-43^M337V^ mESC-MNs

Energy is an essential requirement for powering the molecular motors that drive axonal transport. Previous studies have shown that both glycolysis and mitochondrial respiration have roles in providing energy to power axonal transport of signalling endosomes and mitochondria, respectively [32–34].

**Fig. 5:**
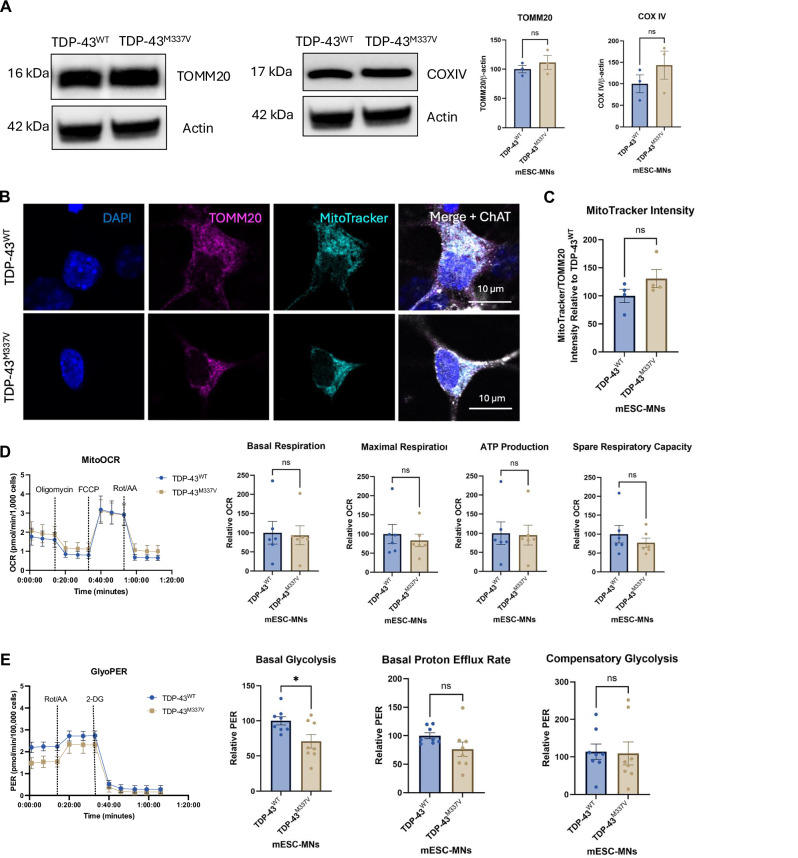
TDP-43^M337V^ mESC-MNs show reduced basal glycolysis but no impairments to mitochondrial respiration. **A** Western blots and quantification of expression levels for TOMM20 and COX IV in TDP-43^WT^ and TDP-43^M337V^ mESC-MNs. **B** Immunocytochemistry for TOMM20, MitoTracker and ChAT. **C** Quantification of MitoTracker intensity normalised to TOMM20 expression in TDP-43^WT^ and TDP-43^M337V^ mESC-MNs. *N* = 4 differentiations. **D** Investigation of mitochondrial respiration in TDP-43^WT^ and TDP-43^M337V^ mESC-MNs using the Seahorse Mito Stress Test. *N* = 6 differentiations. **E** Investigation of glycolysis in TDP-43^WT^ and TDP-43^M337V^ mESC-MNs using the Seahorse Glycolytic Rate Assay. *N* = 8 differentiations. *p = <0.05. Error bars = SEM.

To investigate whether impairments to cellular bioenergetics could contribute to axonal transport deficits in TDP-43^M337V^ mESC-MNs, we first examined mitochondrial number and mitochondrial membrane potential. Western blotting with TOMM20 and COXIV antibodies was performed to assess mitochondrial number, which identified no significant differences between TDP-43^WT^ and TDP-43^M337V^ mESC-MNs (Fig. 5A; TOMM20: *p* = 0.4390; COX IV *p* = 0.3250). To assess mitochondrial membrane potential, immunocytochemistry was performed with MitoTracker^TM^ Red CMXRos, where the uptake of this dye occurs in a membrane potential-dependent manner. Analysis revealed no significant differences between TDP-43^WT^ and TDP-43^M337V^ mESC-MNs (Fig. 5B-C; *p* = 0.1740). We observed no significant differences in mitochondrial shape descriptors measured by analysis of TOMM20, including mitochondrial circularity (*p* = 0.7419) or aspect ratio (*p* = 0.5805) (Supplementary Fig. 3), or in the expression of the mitochondrial fusion protein OPA1 (Supplementary Fig. 4).

We next investigated energy production from mitochondrial respiration and glycolysis in TDP-43^WT^ and TDP-43^M337V^ mESC-MNs using the Seahorse Mito Stress Test and Glycolytic Rate Assay at day 20. TDP-43^M337V^ mESC-MNs showed no significant differences in basal respiration (*p* = 0.8688), maximal respiration (*p* = 0.5887), ATP production (*p* = 0.9042) or spare respiratory capacity (*p* = 0.4073) compared to TDP-43^WT^ controls (Fig. 5D). However, analysis of glycolysis revealed significantly reduced basal glycolysis (*p* = 0.0218) in TDP-43^M337V^ mESC-MNs, with no significant differences in basal proton efflux rate (*p* = 0.1046) or compensatory glycolysis identified (*p* = 0.9111) (Fig. 5E).

## Discussion

In previous work, we have shown that TDP-43^M337V^ mESC-MNs show impaired responses to oxidative stress [14]. Here, we build on this work by demonstrating that in unstressed conditions, TDP-43^M337V^ mESC-MNs demonstrate several disease-relevant phenotypes, including reduced viability, impaired bidirectional axonal transport and reduced glycolysis compared to TDP-43^WT^ controls. These phenotypes occur without clear evidence of TDP-43 cytoplasmic mislocalisation or aggregation, suggesting that mislocalisation may not be driving cellular dysfunction in this model.

Motor neurons possess uniquely long axons relative to other neuronal cell types, and axonal transport is essential for the maintenance of communication between the cell soma and the distal synapse. Given this, disruption of axonal transport provides an attractive theory as to why motor neurons may be selectively vulnerable to degeneration in ALS. Initial evidence for impaired axonal transport in ALS came from human post-mortem studies, where researchers identified abnormal accumulations of cargo within the cell soma and proximal axon, including cytoskeletal components, lysosomes and mitochondria [35–39]. Impaired axonal transport has now been identified as a prominent feature observed across genetically distinct models of ALS, including *SOD1*, *C9orf72*, *FUS*, and *TARDBP* 21–26]. In models of *TARDBP* mutations, impairments to the axonal transport of signalling endosomes, mitochondria and of TDP-43 itself have been reported [25, 27, 40]. Of particular relevance, analysis of axonal transport in our TDP-43^M337V^ BAC transgenic mouse revealed significant impairments to retrograde transport of neurotrophin-containing signalling endosomes [27]. Our findings in the present study recapitulate these impairments in vitro, where TDP-43^M337V^ mESC-MNs expressing the same BAC genetic construct as in the mouse model, demonstrate a reduction in the speed of retrograde transport of CTB-positive signalling endosomes. We were interested to investigate whether these impairments were specific to the transport of CTB-positive signalling endosomes, or if impaired axonal transport of additional cargo, in particular mitochondria, might be observed. We identified significant reductions in the speed of bi-directional mitochondrial transport in TDP-43^M337V^ mESC-MNs compared to controls. These impairments occurred without evidence of TDP-43 mislocalisation or aggregation, in support of previous findings demonstrating that TDP-43 mislocalisation and aggregation is not a prerequisite for disrupted axonal transport [40].

The mechanisms by which TDP-43^M337V^ may lead to disruption of axonal transport remain unclear. Effective axonal transport relies on a stable microtubule cytoskeleton and the abundance and activity of molecular motor proteins and their respective adaptors. Here, we identified a significant reduction in neurite area in TDP-43^M337V^ mESC-MNs. It is possible that this reduced neurite area reflects reduced cell viability, as we also observed a significant reduction in nuclei number. However, it could also indicate reduced microtubule stability. Impairments to the microtubule cytoskeleton have been linked to several neurodegenerative diseases, including ALS. Mutations in the α-tubulin gene, which combines with β-tubulin to form α-β heterodimers, have been identified as a potential cause of ALS in rare cases [41]. Microtubule stability is influenced by intrinsic factors such as tubulin isoforms and post-translational modifications (including acetylation and tyrosination) and extrinsic factors such as the expression and phosphorylation of microtubule-associated proteins (MAPs), including tau, MAP2 and MAP1B [42–45]. Of particular relevance in the context of *TARDBP* mutations, loss of TDP-43 has been shown to reduce levels of the microtubule regulator stathmin-2, leading to reduced neurite outgrowth and axonal regrowth defects [46]. A more detailed understanding of how the TDP-43^M337V^ mutation influences microtubule stability and whether changes in microtubule stability precede reduced viability will help clarify the drivers of reduced neurite area in this model.

Regarding motor protein expression, we observed no significant differences in the expression of anterograde motors (kinesins) or retrograde motor protein complexes (dynein and dynactin). However, it is possible that the activity of the motor proteins or their ability to attach to cargoes or microtubules could be affected. The aberrant phosphorylation of motor proteins by protein kinases has been shown to alter motor activity, motor binding to microtubules/cargo and contribute to axonal transport defects in neurodegenerative diseases [47, 48]. For example, activity of the neuron-specific kinase JNK3 has been shown to impair both anterograde and retrograde axonal transport [49] and cause kinesin-1 to disengage from microtubules [50]. Additionally, GSK3β activity has been shown to disrupt kinesin-1 movement by altering kinesin-1 binding to microtubules and membranes [51]. To gain further insight into the mechanisms driving the bidirectional axonal transport defects observed in this model, it would be interesting to investigate whether the activity of molecular motors and their attachment to cargoes/membranes is disrupted in TDP-43^M337V^ mESC-MNs.

In addition to the structural and functional mediators of axonal transport, movement of motor proteins along axons requires sufficient energy production. In TDP-43^M337V^ mESC-MNs we saw no significant differences in mitochondria number, membrane potential, mitochondrial shape descriptors, or ATP production through mitochondrial respiration. However, TDP-43^M337V^ mESC-MNs demonstrated significantly reduced basal glycolysis compared to controls. Local ATP production via glycolysis has previously been shown to provide energy to power the fast axonal transport of vesicles in *Drosophila* larvae and cortical neurons. Localisation of glycolytic enzymes, including GAPDH, PGK1, PGM, ENO1, ENO2 and PK, to vesicles provided ‘on-board’ energy to drive transport [32, 33]. Given this, reduced basal glycolysis may contribute to the impaired axonal transport of signalling endosomes that we observe in TDP-43^M337V^ mESC-MNs. But reduced glycolysis may not explain the impairments we see to mitochondrial transport, which is primarily driven by ATP produced from the mitochondria themselves [32]. As we identified no significant impairments to mitochondrial ATP production, this suggests that alternative mechanisms may underlie axonal transport deficits. Moreover, TDP-43^M337V^ mESC-MNs demonstrate consistent impairments to axonal transport, affecting the bidirectional transport of two distinct cargoes, suggesting a generalised impairment to global axonal transport in this model.

The TDP-43^M337V^ mutation is located within the C-terminal domain of the protein, which has important roles in its ability to bind to other proteins. Interactions between TDP-43 and other proteins are important for the regulation of key cellular functions, including alternative splicing, translation and protein homoeostasis [52–55]. Changes in the TDP-43^M337V^ protein interactome in basal and oxidative stressed conditions have previously been reported by our group and demonstrate functional significance [4]. In TDP-43^M337V^ mESC-MN and human induced pluripotent stem cell models (hiPSC)-MN models, phenotypes including impaired stress granule formation, disrupted endosomal-extracellular transport, impaired axonal transport and altered cellular bioenergetics have been associated with altered interactions between TDP-43^M337V^ and proteins involved in these pathways [4, 56]. However, the impact of numerous other altered interactions identified through this work, including several interactions between TDP-43^M337V^ and proteins involved in axonal transport and cellular bioenergetics, remain relatively understudied. These include a gain of interaction between TDP-43^M337V^ and the microtubule-related protein tubulin-alpha 1A (TBA1A), as well as loss of interaction between TDP-43^M337V^ and neurofilament medium (NFM), and the actin-binding protein, profilin-1 (PF1), where mutations in the latter have been identified in a small number of ALS cases [57]. Furthermore, a loss of interaction is observed between TDP-43^M337V^ and key glycolytic enzymes PGK1, PGAM1, and ALDOA. Future studies are required to establish whether these interactome changes may be functionally significant and investigate whether interventions that alter the TDP-43 interactome represent a viable therapeutic strategy.

In this work, we provide extensive phenotypic characterisation of the TDP-43^M337V^ mESC-MN model of ALS. We find that low-level expression of the TDP-43^M337V^ mutation is sufficient to impair cellular viability, axonal transport and glycolysis. As these phenotypes occur without evidence of TDP-43 mislocalisation or aggregation, our findings suggest that overt TDP-43 mislocalisation is not a prerequisite for cellular dysfunction in this model. Although further clarification around the mechanisms underlying these phenotypes is required, this study provides valuable insight into the key cellular pathways altered by the TDP-43^M337V^ mutation that warrant further investigation as potential therapeutic targets.

## Supplementary information

Supplementary Figure 1

Supplementary Figure 2

Supplementary Figure 3

Supplementary Figure 4

Full and uncropped Western blots

## Data Availability

The datasets generated and analysed in this study are available from the corresponding author upon reasonable request.
