# Supplementary figures and images for: Mutant TDP-43 drives impairments in axonal transport and glycolysis in a mouse stem-cell-derived motor neuron model of amyotrophic lateral sclerosis (ALS)
Source: Cell Death Dis. 2026 Jan 31;17(1):193. doi: 10.1038/s41419-026-08437-2 (PMC12877184; doi:10.1038/s41419-026-08437-2)

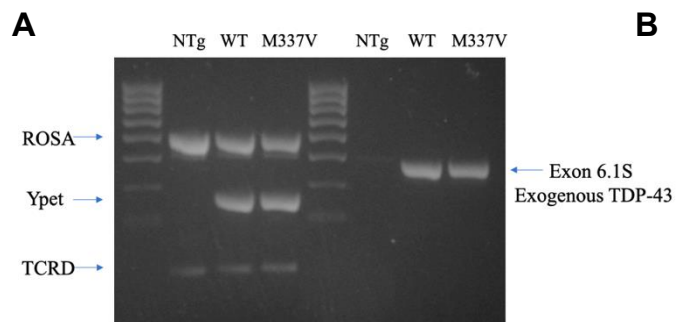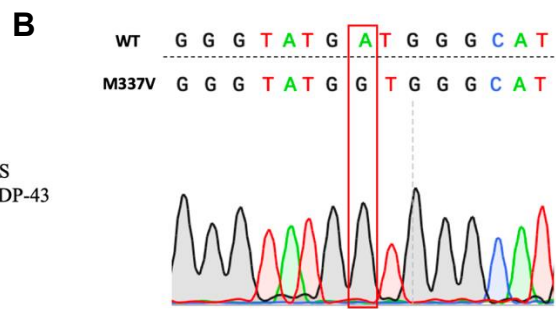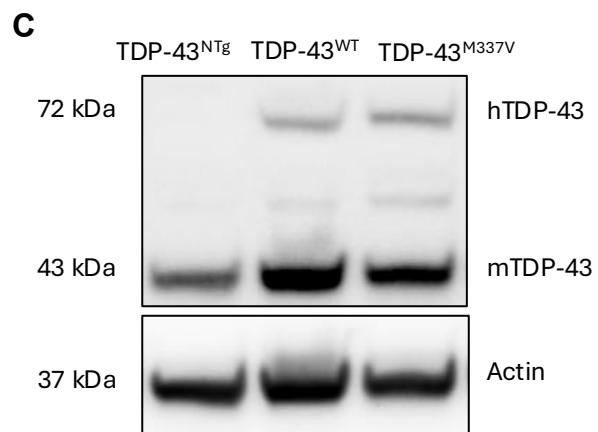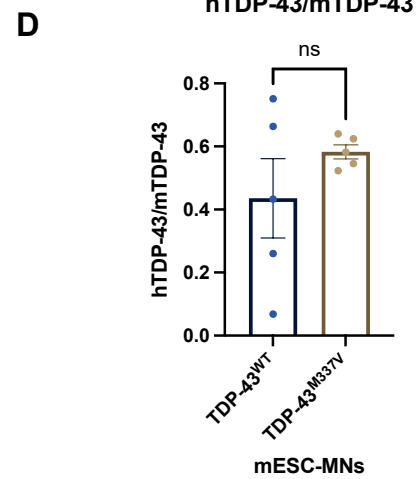

Supplement: Supplementary file 1 — Supplementary Figure 1 [file 41419_2026_8437_MOESM1_ESM.pdf]

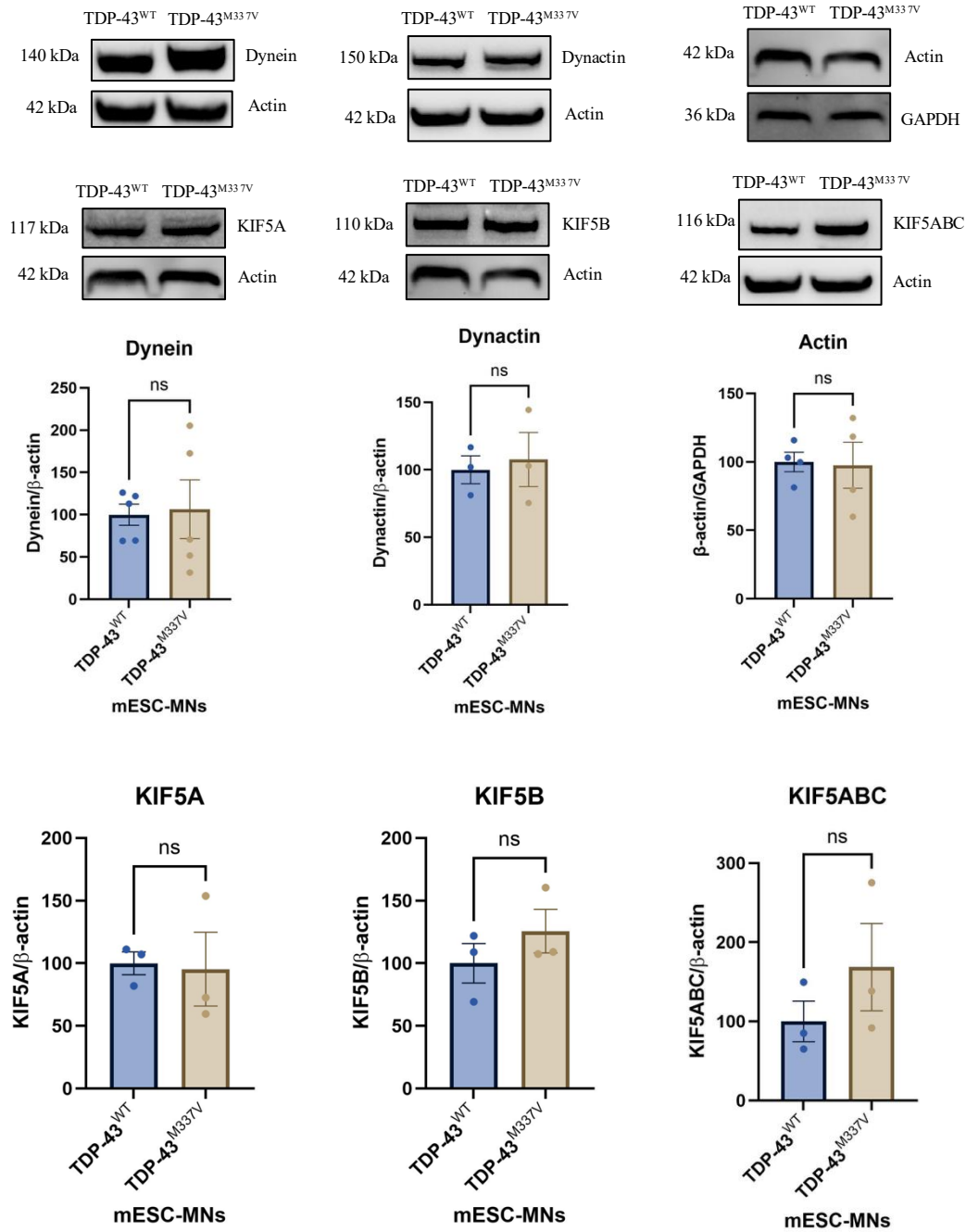

Supplement: Supplementary file 2 — Supplementary Figure 2 [file 41419_2026_8437_MOESM2_ESM.pdf]

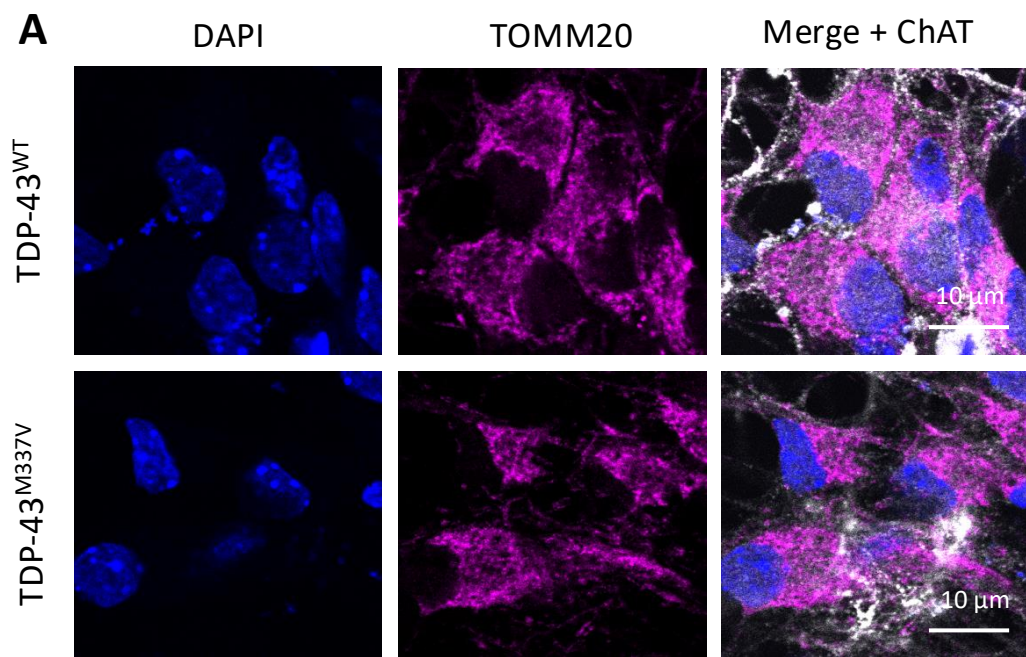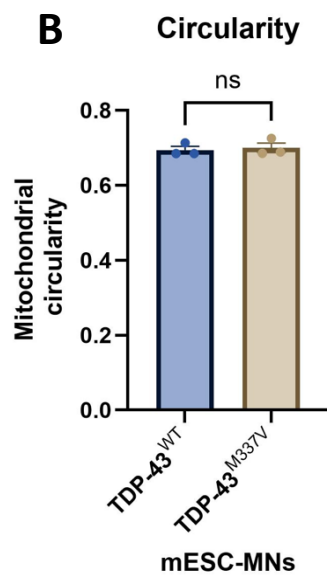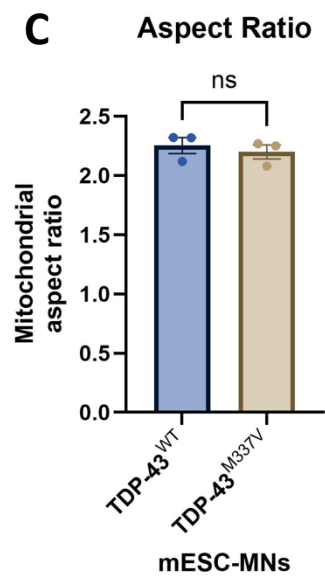

Supplement: Supplementary file 3 — Supplementary Figure 3 [file 41419_2026_8437_MOESM3_ESM.pdf]

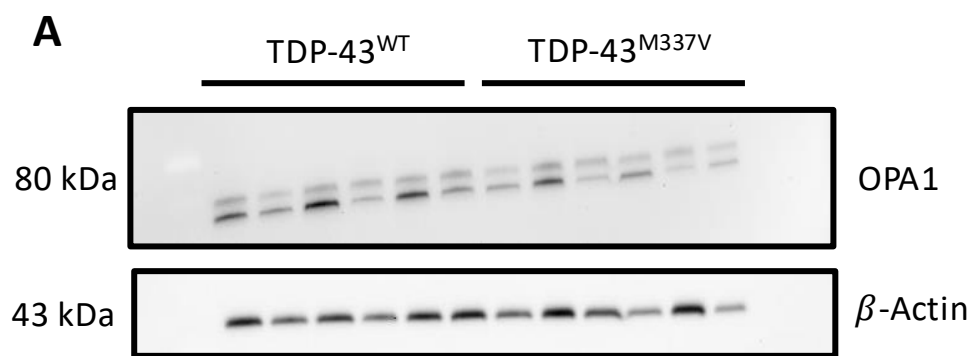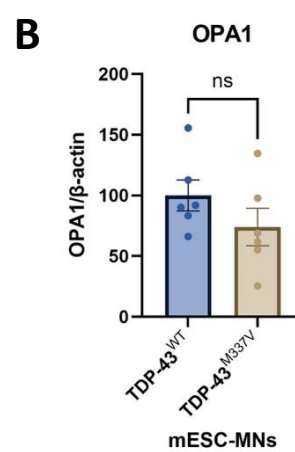

Supplement: Supplementary file 4 — Supplementary Figure 4 [file 41419_2026_8437_MOESM4_ESM.pdf]
