# Supplementary material for: Mutant TDP-43 drives impairments in axonal transport and glycolysis in a mouse stem-cell-derived motor neuron model of amyotrophic lateral sclerosis (ALS)
Source: Cell Death Dis. 2026 Jan 31;17(1):193. doi: 10.1038/s41419-026-08437-2 (PMC12877184; doi:10.1038/s41419-026-08437-2)
Supplement: Supplementary file 5 — Full and uncropped Western blots [file 41419_2026_8437_MOESM5_ESM.pdf]

Full length uncropped western blots – TDP-43

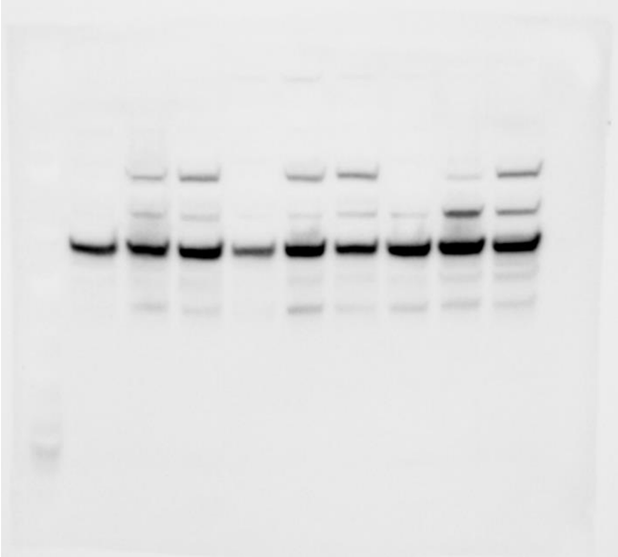

TDP-43: Non transgenic, TDP-43^{WT}, and TDP-43^{M337V}, n = 3

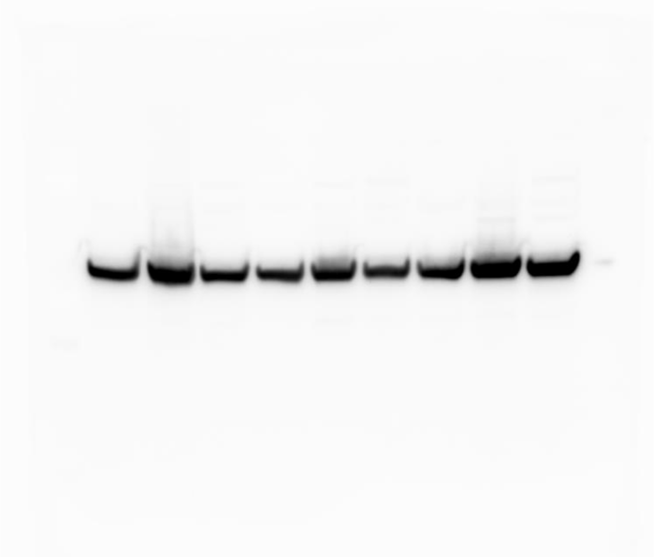

Actin: Non transgenic, TDP-43^{WT}, and TDP-43^{M337V}, n=3

Full length uncropped western blots – axonal transport KIF5A

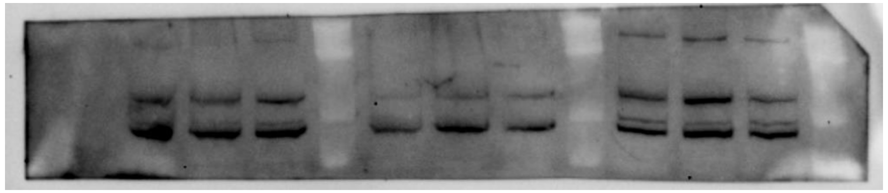

KIF5A: Non transgenic, TDP-43^{WT}, and TDP-43^{M337V}, n =3

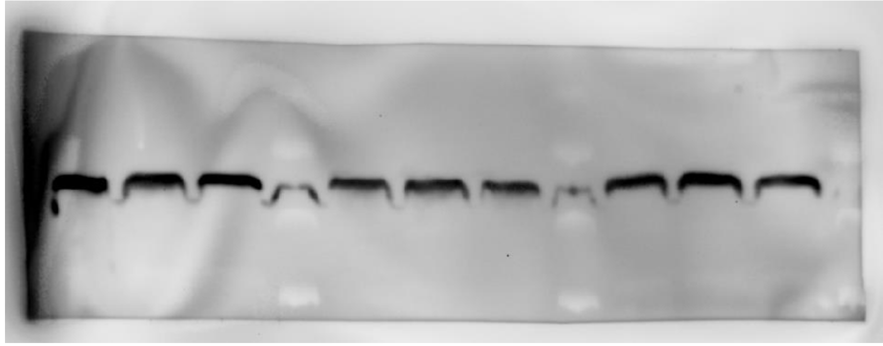

Actin: Non transgenic, TDP-43^{WT}, and TDP-43^{M337V}, n=3

Full length uncropped western blots – axonal transport KIF5B

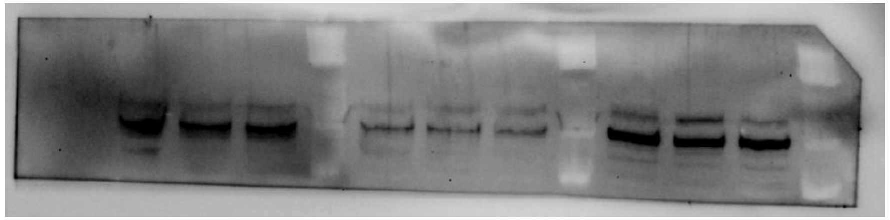

KIF5B: Non transgenic, TDP-43^{WT}, and TDP-43^{M337V}, n =3

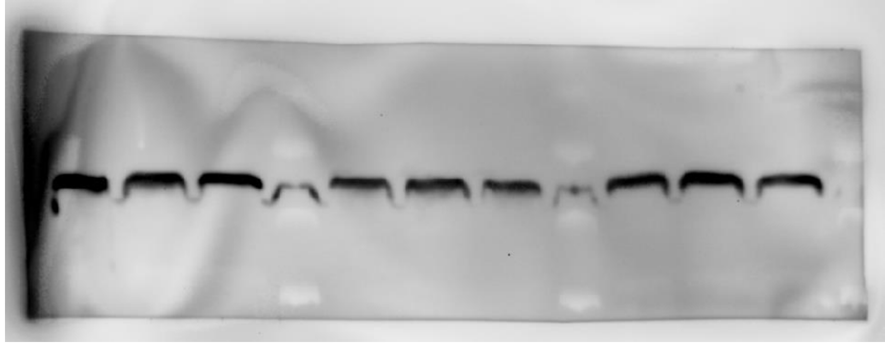

Actin: Non transgenic, TDP-43^{WT}, and TDP-43^{M337V}, n =3

Full length uncropped western blots – axonal transport KIF5ABC

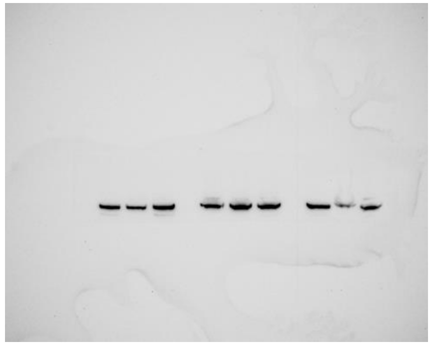

KIF5ABC: Non transgenic, TDP-43^{WT}, and TDP-43^{M337V}, n =3

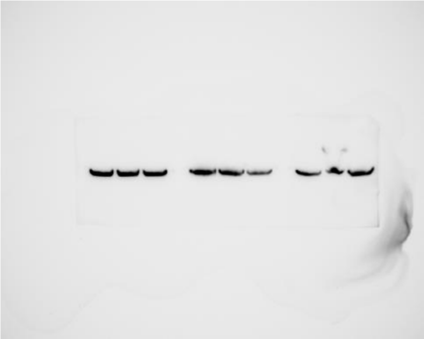

Actin: Non transgenic, TDP-43^{WT}, and TDP-43^{M337V}, n =3

Dynactin

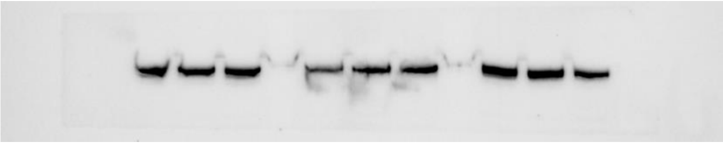

Dynactin: Non transgenic, TDP-43^{WT}, and TDP-43^{M337V}, n =3

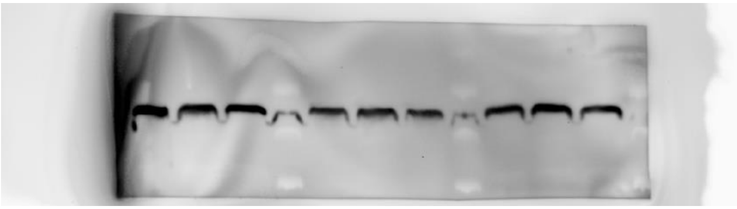

Actin: Non transgenic, TDP-43^{WT}, and TDP-43^{M337V}, n =3

Dynein

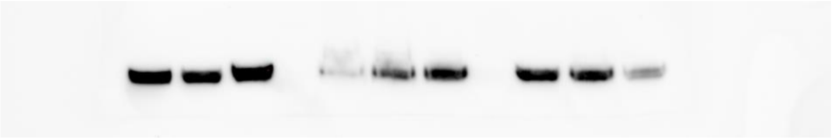

Dynein: Non transgenic, TDP-43^{WT}, and TDP-43^{M337V}, n =3

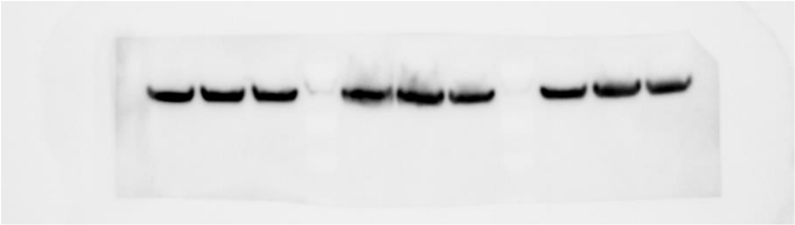

Actin: Non transgenic, TDP-43^{WT}, and TDP-43^{M337V}, n =3

TOMM20

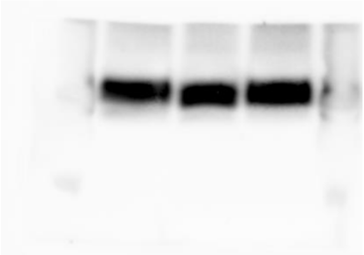

TOMM20: Non transgenic, TDP-43^{WT}, and TDP-43^{M337V}

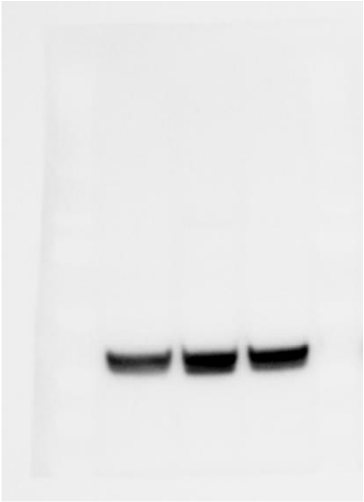

Actin: Non transgenic, TDP-43^{WT}, and TDP-43^{M337V}

COXIV

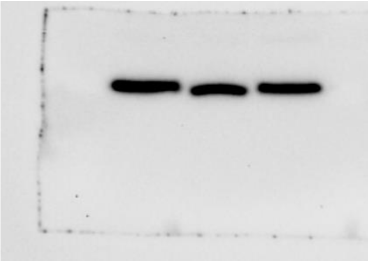

COX IV: Non transgenic, TDP-43^{WT}, and TDP-43^{M337V}

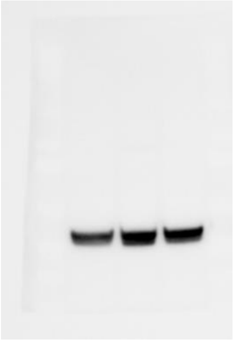

Actin: Non transgenic, TDP-43^{WT}, and TDP-43^{M337V}

OPA1

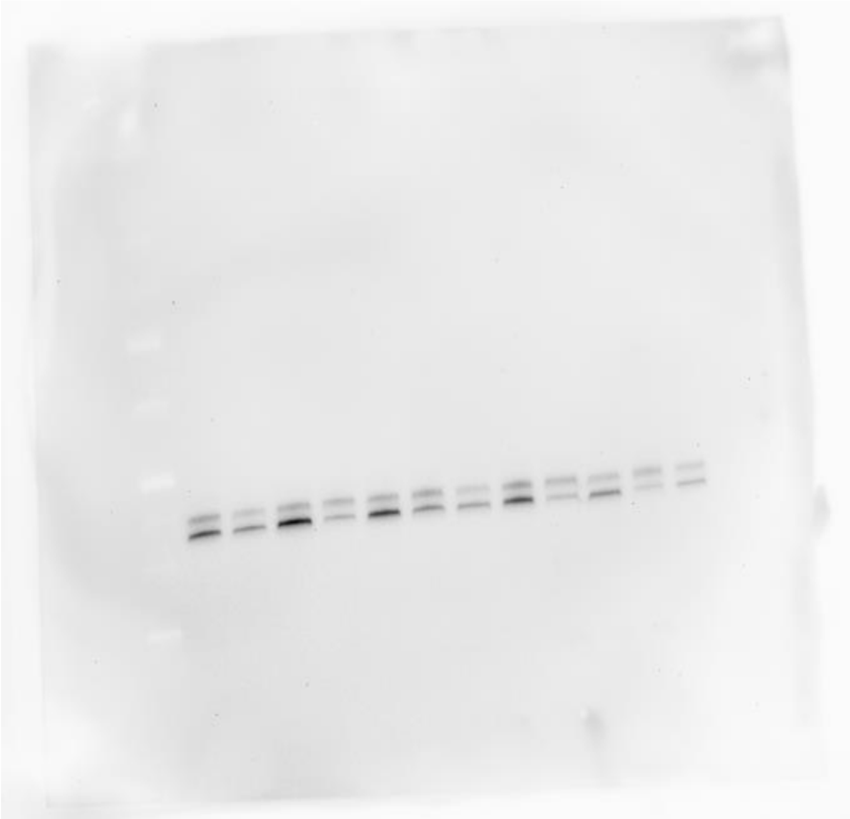

OPA-1: TDP-43^{WT} (lanes 1-6) and TDP-43^{M337V} (lanes 7-13) n=6

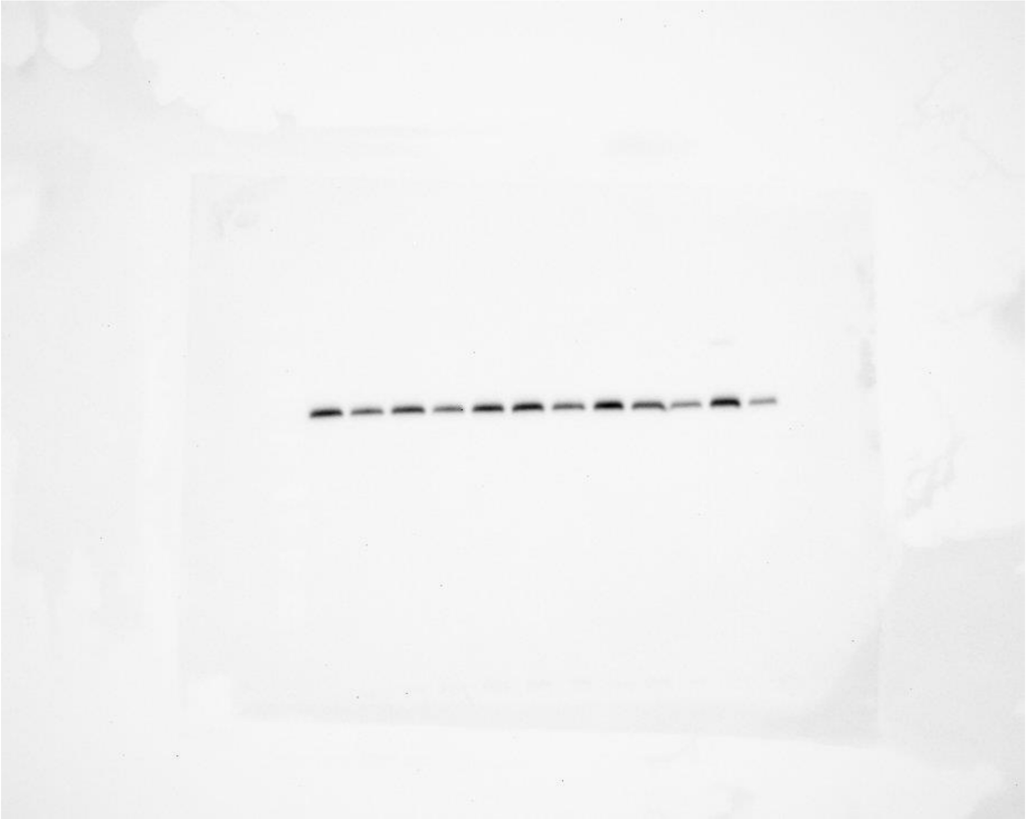

Actin: TDP-43^{WT} (lanes 1-6) and TDP-43^{M337V} (lanes 7-13) n=6
